# May the palps be with you – new insights into the evolutionary origin of anterior appendages in Terebelliformia (Annelida)

**DOI:** 10.1186/s40850-021-00094-6

**Published:** 2021-11-16

**Authors:** Paul Kalke, Patrick Beckers, Conrad Helm

**Affiliations:** 1 Department of Animal Evolution and Biodiversity, University of Goettingen, Goettingen, Germany; 2 Institute of Evolutionary Biology and Ecology, University of Bonn, Bonn, Germany

**Keywords:** Segmented worms, Morphology, Nervous system, Tentacles, Comparative approach, Histology, cLSM

## Abstract

**Background:**

Head appendages in Annelida contribute significantly to the immense morphological diversity in this spiralian taxon. Nevertheless, the evolutionary origin of annelid antennae, palps, cirri and tentacles are part of vast theories and debates that took place over decades. One of these heavily discussed groups are the Terebelliformia, which bear numerous anterior tentacles originating from different regions of the head. The question, whether these tentacles are homologous to feeding palps in other annelids or if these structures evolved convergently in terebellids and the remaining taxa, has been highly debated in the past.

**Results:**

By using morphological methods including immunohistochemistry, confocal microscopy, Azan-stained serial sections and 3D-visualisation, we are able to shed new light and a fresh look on the old question of the evolutionary origin of the buccal tentacles and their associated head structures in Terebelliformia. Our investigations show that the brains of the ampharetid *Hypania invalida* and the aulophora larvae of *Lanice conchilega* (Terebellidae) consist of a dorsal, more prominent and a more slender, ventral brain region. Neurite bundles innervating the buccal tentacles split off from the ventral and dorsal root within the ventral brain region and thus originate from the dorsal and ventral root of the circumoesophageal connectives. Hence, the observed neurite bundles fulfil the morphological criteria for the innervating neurite bundles of feeding palps known from Paleoannelida.

**Conclusions:**

We disagree with former conclusions that buccal tentacles are part of the alimentary canal. Based on the presented data, the buccal tentacles of terebelliform taxa are innervated by neurite bundles and can be homologized with peristomial feeding palps of other Annelida.

Our comparative investigations reveal important insights into morphological changes during the evolution of anterior head appendages in Terebelliformia and Annelida in general. Nevertheless, our analyses also illustrate the gaps in knowledge and that more investigations throughout the annelid tree are necessary to explain and understand the huge diversity of annelid anterior appendages.

## Background

Annelida represent a fascinating and diverse group of invertebrates. In particular, the frontal end shows a variety of head appendages in terms of shape, function and number, and contributes to this immense diversity of forms within segmented worms. A remarkable and highly discussed annelid taxon in this respect is Terebelliformia. The exact phylogenetic position of terebelliform lineages and the topology within the taxon were under persistent discussion for a long time [1–3]. However, phylogenomic approaches placed Terebelliformia confidently within the Sedentaria and hence they represent the sister taxon to the Maldanomorpha (Maldanidae + Arenicolidae) [4, 5]. Recently, new analyses uncovered the phylogenetic relations within the group, and suggest a taxon arrangement of Ampharetidae + Alvinellidae and Trichobranchidae + Terebellidae (including Melinnidae) [6]. Furthermore, the authors present Pectinariidae as sistergroup to all other Terebelliformia and therefore recover the idea of an “archaeoterebellomorph” with four pairs of branchiae and a large prostomium [1].

**Fig. 1:**
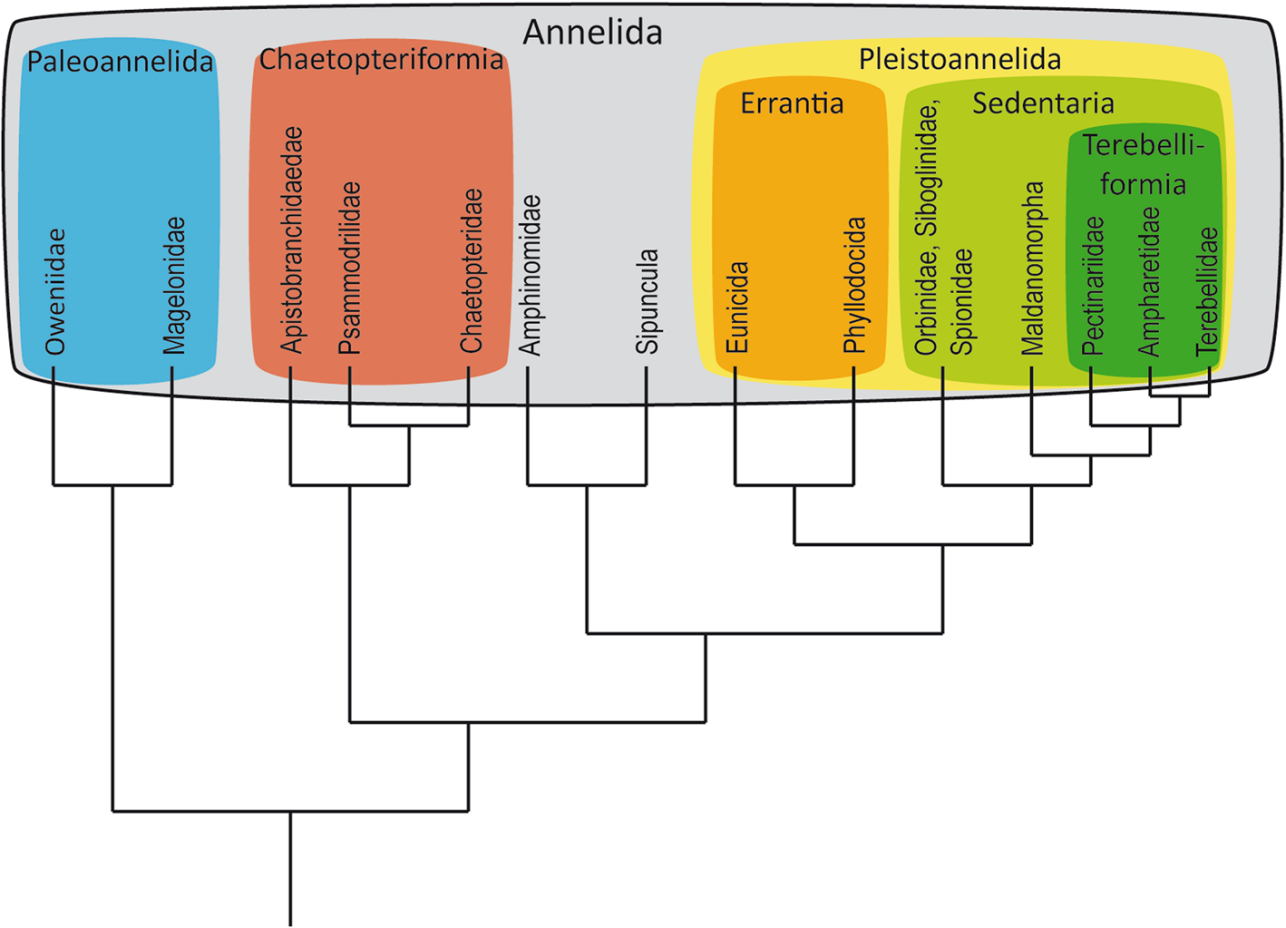
Combined and simplified topology showing recent hypotheses concerning relationships within Annelida. The scheme is based on different sources [5–7]

Deeply nested within the sedentary annelids [5], see also Fig. 1), most members of the Terebelliformia bear numerous, well-developed anterior head appendages.

Besides highly diverse branchial structures, numerous slender buccal tentacles can be observed throughout the family. Notably, these buccal tentacles and their evolutionary origin are part of a long-lasting and still ongoing discussion [1, 8–14].

The position of these buccal tentacles differs inside Terebelliformia and reaches from tentacles surrounding the mouth opening in an arc-like frame (in Pectinariidae), over appendages originating from an eversible pharynx (in Ampharetidae) to tentacles located in a dorsal position on the anterior end (in Terebellidae and Trichobranchidae) [6, 12]. So far, two contradictory theories about the evolutionary origin of these buccal tentacles in Terebelliformia are debated:

1. the buccal tentacles are homologous to feeding palps observed in other annelids [8, 9, 11, 12] or
2. the buccal tentacles evolved from buccal structures and therefore represent a convergently evolved structure non-homologous to sensory or feeding palps of other Annelida [1, 13, 14].

In the early twentieth century, these terebelliform buccal tentacles and their associated structures were homologised with almost any kind of anterior appendage, even being rudiments of antennae [8, 9]. On the other hand, Orrhage [13] described a ribbon-like, simple brain for Terebelliformia and suggested the buccal tentacles as being an outgrowth of the upper lip. According to his descriptions, the tentacular nerves are closely associated with nerves of the alimentary canal, thus being part of it. In contrast, Rouse and Fauchald [11] suggested that the buccal tentacles of Terebelliformia are homologous to the palps of e.g., Sabellidae and Spionidae - a statement which also follows their Canalipalpata-Aciculata theory. Other authors used the buccal morphology to accentuate the homology to palps by the absence of pharyngeal ciliated fields on tentacles and their area of attachment [12]. Based on these investigations, the terebelliform tentacles are not part of the alimentary canal. Furthermore, developmental studies favour the homology with feeding palps due to the independent maturation of pharyngeal structures and buccal tentacles [15–17].

To shed new light on this long-lasting discussion, and to unveil further details helpful for our understanding of the evolutionary origin of terebelliform anterior appendages, we used a comparative and integrative approach. Our analyses include a variety of morphological methods such as immunohistochemistry, serial, Azan-stained, sections and 3D-visualization. Our comprehensive investigations provide a fresh look on a more than a hundred years ongoing discussion and uncover the evolutionary origin and homology hypotheses for anterior body appendages in terebellids and allies. Additionally, our data will critically challenge existing knowledge dealing with this topic and contribute to our understanding concerning the evolution of character complexes in annelid worms.

## Results

**Fig. 2:**
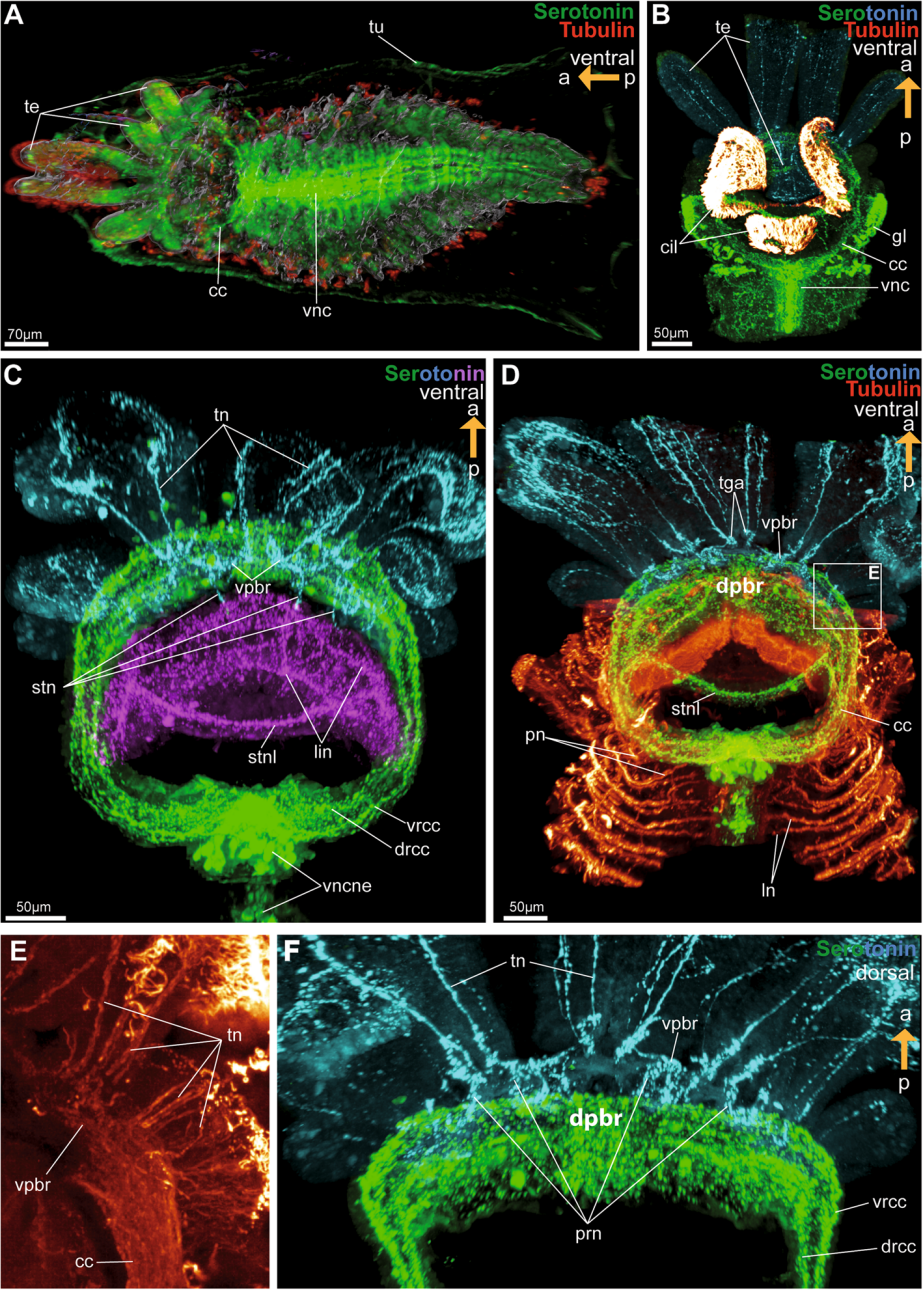
Nervous system of the anterior region of aulophora larvae of *Lanice conchilega*, cLSM micrographs. **A**. General anatomy of aulophora larvae sitting in its tube, overall serotonergic nervous system highlighted in green and α-tubulinergic setae in red. **B**. Serotonergic nervous system anterior region. Central nervous system like brain and vnc are highlighted in green, tentacular nerves in cyan and α-tubulinergic cilia on the lateral and lower lips in red. **C**. Close-up of the serotonergic nervous system of the head region. Ventral region of the brain and tentacular nerves are highlighted in cyan, dorsal region of the brain, cc and vnc in green and stomatogastric nerves including lip nerves in magenta. **D**. General anatomy of the serotonergic and α-tubulinergic nervous system of the anterior region. Ventral region of the brain and tentacular nerves are highlighted in cyan, central and stomatogastric nervous system in green and α-tubulinergic nervous system in red. **E**. Close-up of micrograph D. α-tubulinergic nervous system in red. On the right side innervation of a developing palp is shown. Additionally, displayed is the transition of cc to the ventral brain region. **F**. Close-up of the brain region, showing the prostomial nerves connecting the dorsal with the ventral brain region. cc – circumoesophageal connectives; cil – cilia of lips; dpbr – dorsal region of brain; drcc – dorsal root of cc; gl – glandular cells; ln – lateral nerves; pn – peripheral nerves; prn – prostomial nerves; stnl – stomatogastric nerve loop; te – buccal tentacles; tga – tentacular ganglion; tn – tentacular nerves; tu – tube; vncne – neurons of vnc, vpbr – ventral region of brain, vrcc – ventral root of cc

**Fig. 3:**
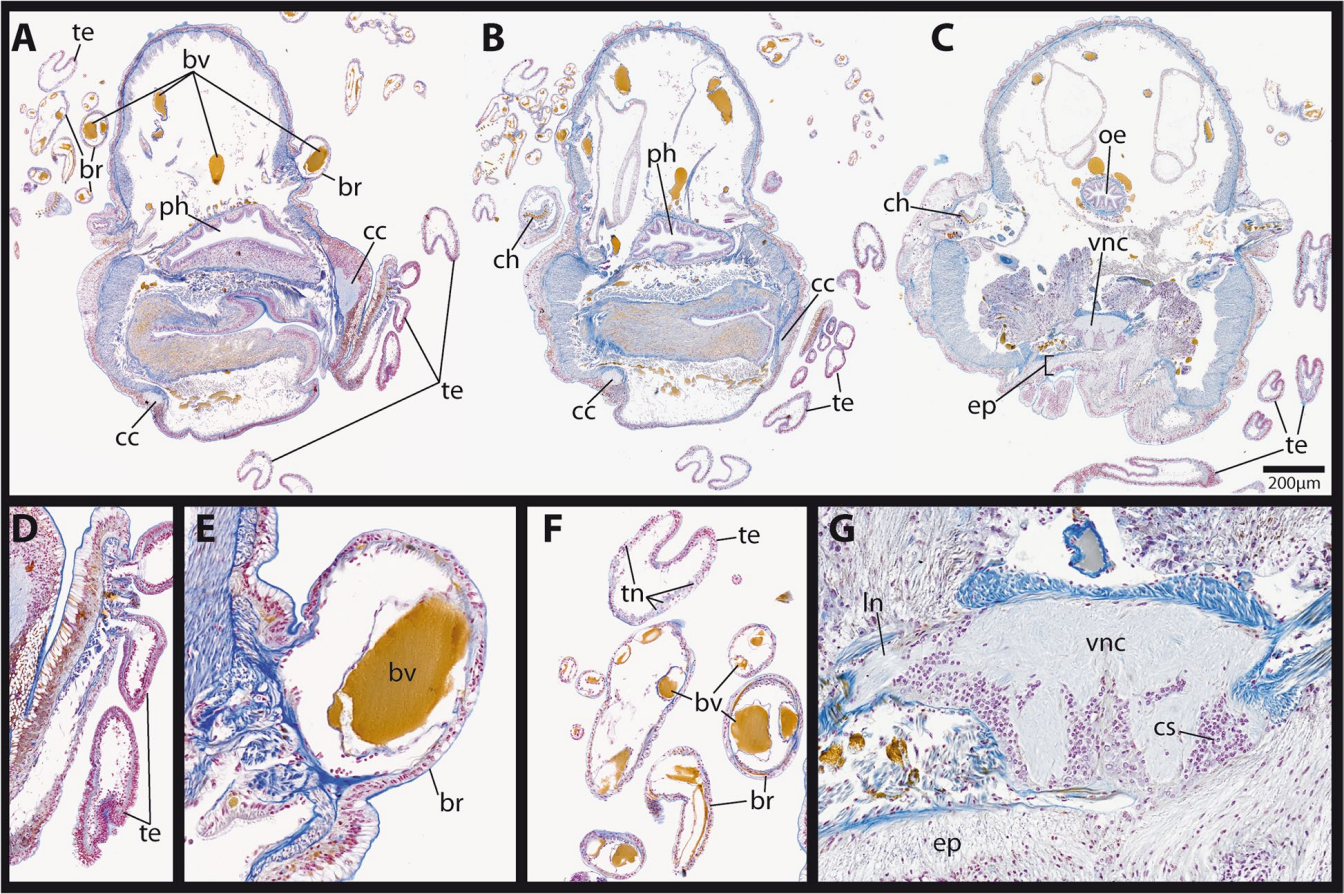
General anatomy of the anterior end of adult *Terebella lapidaria* (Linnaeus 1767), 5 μm sections, Azan-staining, light microscopic images. **A.** Cross-section through anterior head region from which tentacles and branchiae emerge. **B.** Cross-section through middle head region. **C.** Cross-section through posterior head region, cc united to the vnc and anterior oesophageal cavity is identifiable. **D.** Close-up of the cross-section through the lateral origin of buccal tentacles. **E.** Close-up of the cross-section of the dorsal origin of the branchiae. **F.** Close-up of the cross-section through tentacles and branchiae. **G.** Close-up of the cross-section through the ventral nerve cord, huge glandular epidermal cells and the subepidermal vnc are shown. br – branchiae; bv – blood vessel; cc – circumoesophageal connectives; ch – chaete; cs – cell soma; ep – epidermis; ln – lateral nerve; oe – oesophagus; ph – pharynx; te – buccal tentacles; tn – tentacular nerves; vnc – ventral nerve cord

**Fig. 4:**
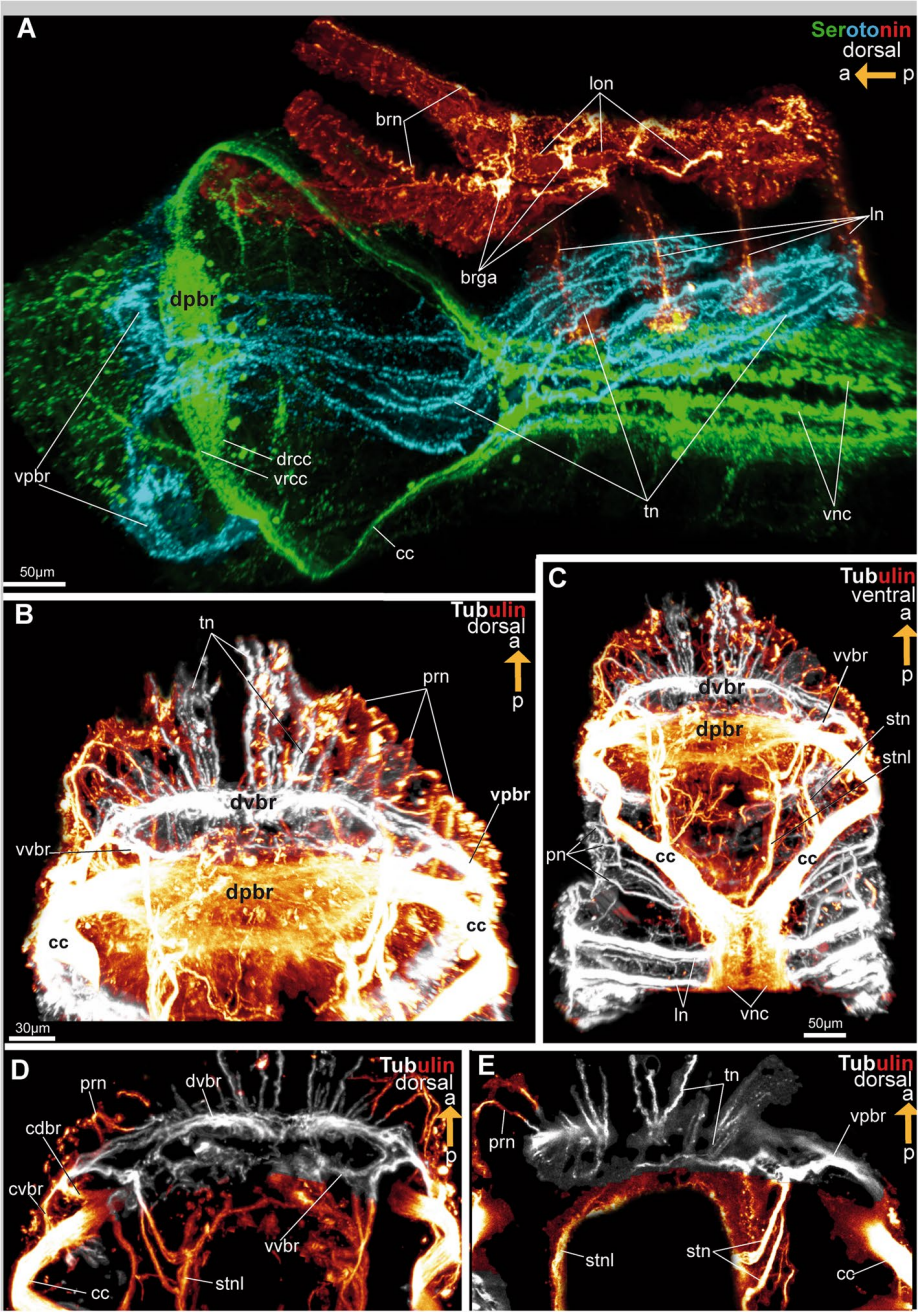
Anterior nervous system of adult *Hypania invalida*, cLSM micrographs. **A.** General anatomy of the serotonergic nervous system. The central nervous is highlighted in green, ventral region of brain and tentacle nerves in blue and branchial neurites in red. **B.** Close-up of brain region stained against acetylated α-tubulin. Ventral region of brain and tentacular nerves highlighted in white. **C.** General anatomy of the tubulinergic nervous system. Ventral region of brain, tentacular nerves and laterally branching nerves are highlighted in white. **D.** Close-up of the ventral brain region stained against α-tubulin, ventral brain region highlighted in white. Connectives of the ventral brain region to the dorsal brain region, the *cc* and the stomatogastric nerve loop are shown. **E.** Close-up of the ventral brain region stained against α-tubulin, ventral brain region highlighted in white. Tentacular nerve originate from both roots of the ventral brain region. Brga – ganglion of branchiae; brn – branchae nerves; cc – circumoesophageal connective; cdbr – connective of dpbr; cvbr – connective of vpbr; dpbr – dorsal region of brain; drcc – dorsal root of cc; dvbr – dorsal root of vpbr; ln – lateral nerves; lon – lateral organ nerve; pn – peripheral nerves; prn – prostomial nerves; stn – stomatogastric nerve; stnl – stomatogastric nerve loop; tn – tentacular nerves; vnc – ventral nerve cord; vpbr – ventral region of brain; vrcc – ventral root of cc; vvbr – ventral root of vpbr

**Fig. 5:**
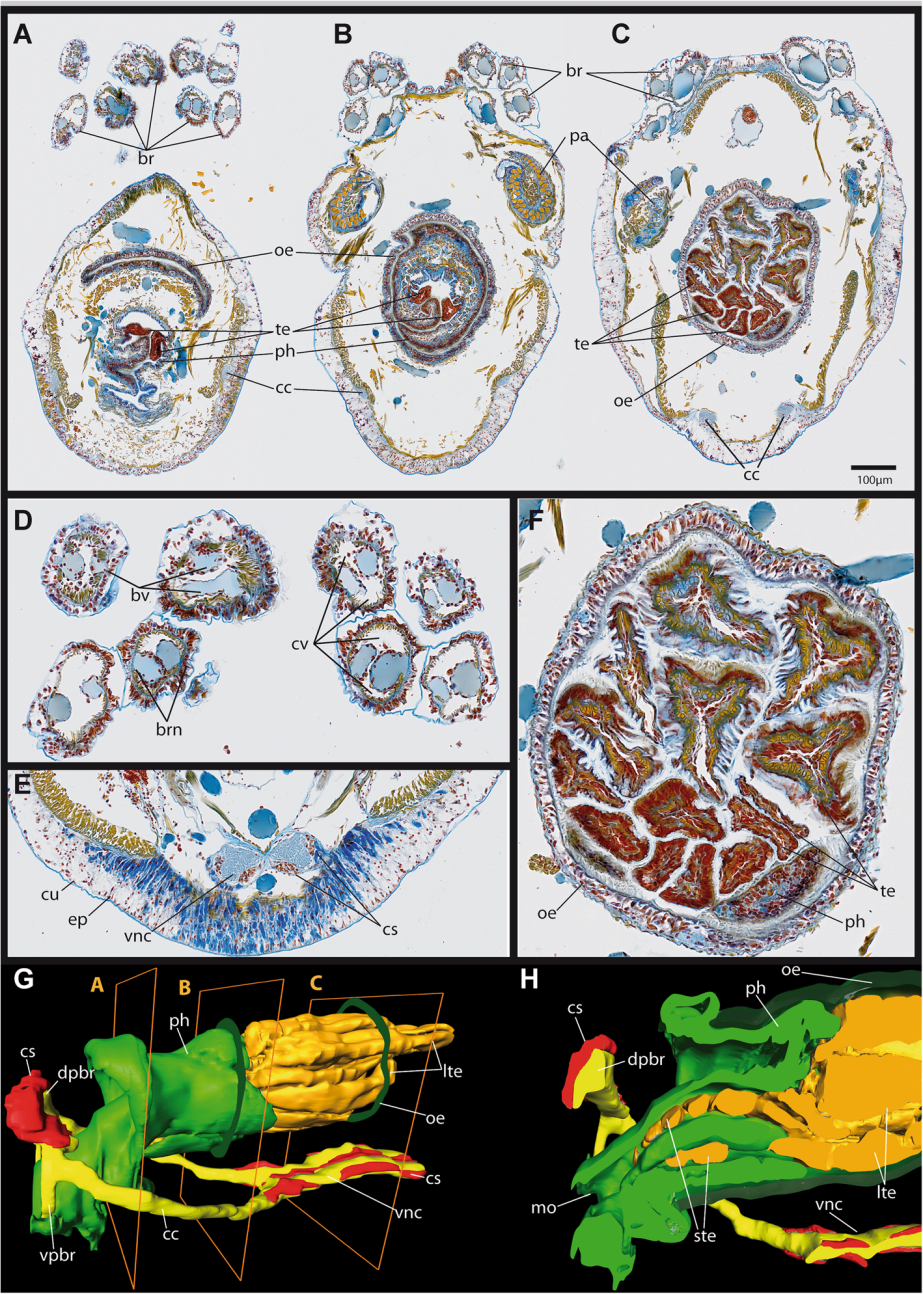
General anatomy of the anterior end of adult *Hypania invalida*, 5 μm sections, Azan-staining, light microscopic images. **A.** Cross-section through anterior part of inverted pharynx, see Fig. G. Short buccal tentacles situated inside the pharyngeal cavity. **B.** Cross-section through the posterior part of the inverted pharynx, see Fig. G. Folded pharynx located inside the oesophageal cavity associated with short tentacles. **C.** Cross-section through the posterior part of the oesophageal cavity, see Fig. G. Long buccal tentacles filling the whole cavity of the oesophagus. **D.** Close-up of a cross-section through the branchiae with two prominent blood vessels, see Fig. A. **E.** Close-up of the anterior region of the subepidermal ventral nerve cord and ganglionic cell somata. **F.** Close-up of the posterior part of the oesophagus, see Fig. C, with numerous long tentacles located inside the oesophageal cavity. **G.** 3D-visualization of the anterior central nervous system and stomatogastric region, long distal buccal tentacle associated with the pharynx, oesophagus omitted. Frames show area of Parafin-sections of Fig. A, B and C. **H.** Digital sagittal section through the 3D-visualisation of the anterior central nervous system and the short buccal tentacles located in the middle part of the pharynx. br – branchiae; brn – branchiael nerves; bv – blood vessel; cc – circumoesophageal connectives; cs – cell soma; cu – cuticle; cv – coelom cavity; dpbr – dorsal region of brain; ep – epidermis; lte – long buccal tentacles; mo – mouth opening; oe – oesophagus; pa – palae bristles; ph – pharynx; ste – short buccal tentacles; te – buccal tentacles; vnc – ventral nerve cord; vpbr – ventral region of brain

**Fig. 6:**
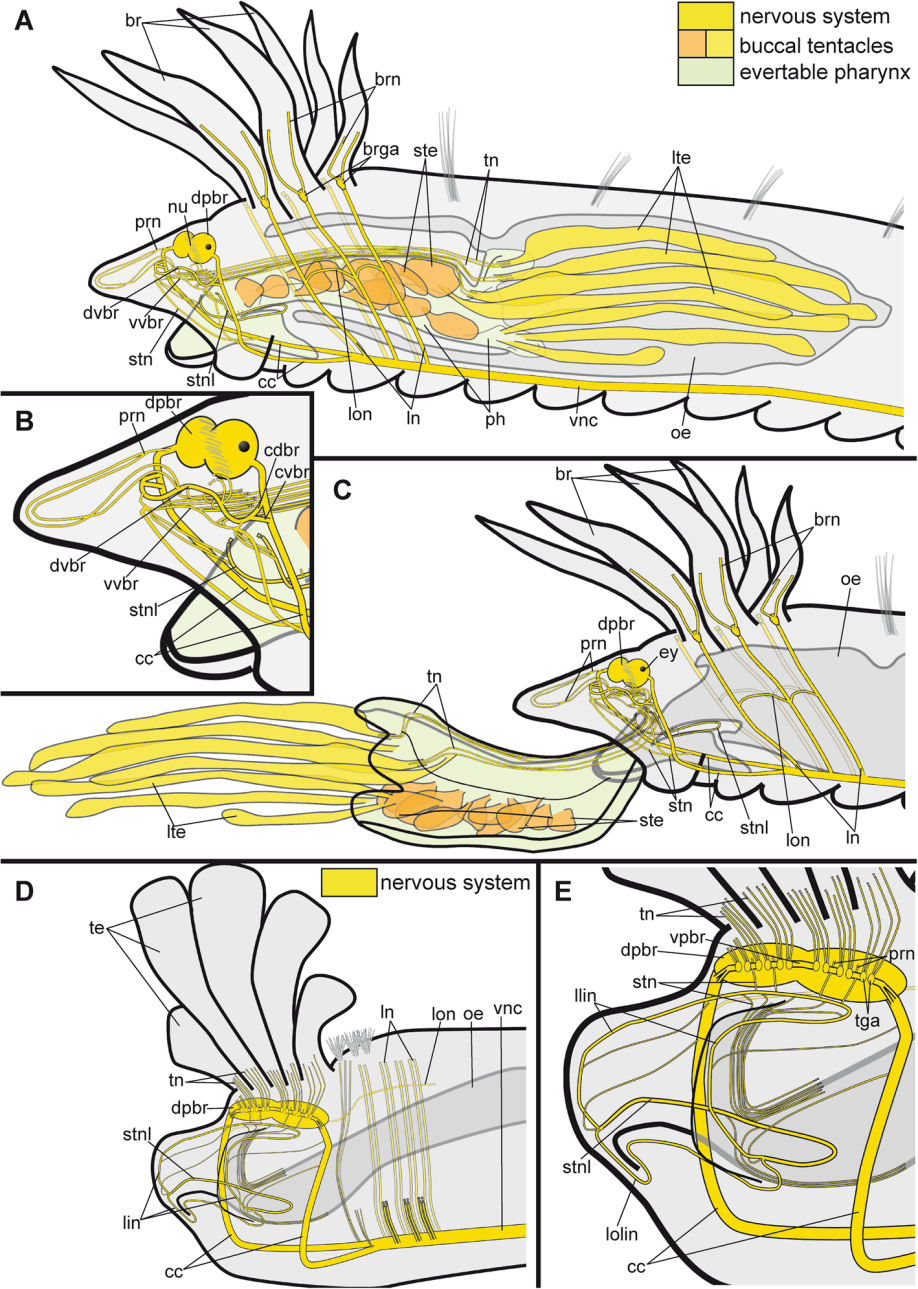
Comparative schematic drawings of the overall nervous system of adult *Hypania invalida* (a-c) and aulophora larvae of *Lanice conchilega* (d, e). Nervous system is yellow, short buccal tentacles are orange and long buccal tentacles are colored in light yellow, the evertable pharynx is colored in mint green, see legend. **A.** Drawing of the anterior region of adult *Hypania invalida*, pharynx in inverted position. **B.** Close-up of the head region, see Fig. A. **C.** Drawing of the anterior region of adult *Hypania invalida*, pharynx in everted position. **D.** Drawing of the anterior region of aulophora larvae of *Lanice conchilega*. **E.** Close-up of the head region, see Fig. D. brga – ganglion of branchiae; br – branchiae; brn – branchiael nerves; cc – circumoesophageal connectives; cdbr – connective of dpbr; cvbr – connective of vpbr; dpbr – dorsal region of brain; ey – eye; llin – lateral lip nerves; lin – lip nerves; ln – lateral nerves; lolin – lower lip nerve; lon – lateral organ nerve; lte – long buccal tentacles; nu – nuchal organ; oe – oesophagus; ph – pharynx; prn – prostomial nerves; ste – short buccal tentacles; stnl – stomatogastric nerve loop; te - buccal tentacle; tn – tentacle nerves; tga – tentacular ganglion; vnc – ventral nerve cord, dvbr – dorsal root of vpbr; vvbr – ventral root of vpbr

To understand the development and anatomy of the head appendages in Terebelliformia, we used aulophora larvae of *Lanice conchilega* (Pallas, 1766) for immunohistochemistry (Fig. 2) and *Terebella labidaria* (Linnaeus, 1767) for Azan-histology (Fig. 3) (as members of the Terebellidae). Furthermore, we investigated adult *Hypania invalida* (Grube, 1860) as a member of the Ampharetidae with both methods (Figs. 4, 5) and summarized all findings in schematic drawings (Fig. 6). In our descriptions of the nervous system, we refer to [18] for used terms and annotations. The description of the head parts follows, if not stated otherwise, [12].

### Central nervous system

#### Aulophora larvae of *Lanice conchilega* and adult *Terebella lapidaria* (Terebellidae)

The aulophora larvae of *Lanice conchilega* already show all features of adult Terebellidae, although the number of anterior tentacles is much less (Fig. 2A). The brain of *L. conchilega* consists of one more prominent dorsal brain region and the delicate anlage of the ventral one (Figs. 2C-F; 6D, E). Both are connected to both roots of the circumoesophageal connective (*cc*) (Figs. 2C, E, F). The ventral brain region protrudes slightly anteriorly from the dorsal one, which is well shown by confocal data but hardly perceptible in Azan-sections. Additionally, several thin longitudinal prostomial nerves connect the dorsal to the ventral region of the brain (Fig. 2F). The subepidermal ventral nerve cord is composed of two longitudinal neurite bundles. Furthermore, four pairs of serotonergic neurite bundles are visible within the ventral nerve cord (*vnc*) of aulophora larvae (Figs. 2A, B). Ventrally, in particular where the *cc* originate, groups of serotonergic cell somata form prominent aggregations (Figs. 2C, D). At regular intervals, two pairs of lateral nerves originate from the *vnc* and proceed in dorsal direction. Although, we could not trace these nerves to their final destination they seems to innervate regular emerging dorsal notopodial structures, such as chaetal muscles or branchiae (in posterior segments). Similar to *H. invalida*, four pair of nerves emerge from the *cc* and proceed in lateral direction (Fig. 2D). They branch off and innervate two lateral epidermal, serotonergic protrusions next to the heavily ciliated lateral lips (Fig. 2B). Glandular cells often catch antibodies. Hence, a strong serotonergic signal without a nervous system specific pattern in huge epidermal cells hints towards the presence of glandular structures in this region (Figs. 3C, G). The stomatogastric nervous system is composed of a lateral lip nerve (Figs. 2C; 6D, E) and a lower lip nerve (Fig. 6D, E). Both are connected to a loop-like stomatogastric nerve (Figs. 2C, D; 6D, E). The lateral lip nerve is connected to the ventral region of the brain via several thin stomatogastric nerves (Figs. 2C; 6E). Along the nerves of the lateral lip, a dense meshwork of slender neurite bundles is coating the upper mouth region (see Fig. 2C). Additionally, numerous delicate neurite bundles originate at the lateral lip nerve and proceed along the dorsal side of the pharynx as found for the ventral side of the stomatogastric loop-like nerve as well (see Figs. 6D, E). The lip nerves and the stomatogastric loop-like nerve are connected to the *cc* via few thin neurite bundles (see Figs. 6D, E).

#### *Hypania invalida* (Ampharetidae)

The brain of *Hypania invalida* is located subepidermally. It consists of a dorsal and ventral region similarly arranged as described for *L. conchilega* (Figs. 4A-C; 6A-C). The dorsal region of the brain (dpbr) is the more prominent part (Figs. 4A-C), linked with numerous cell bodies located dorsally to the brain. The more delicate ventral region of the brain (vpbr), better perceptible in confocal data than in Azan-sections, show a more advanced protrusion from the dorsal region compared to *L. conchilega*. It is connected to the circumesophageal connectives (cc) via a ventral brain connective (cvbr) (Fig. 4D). Additionally, it is connected to the dorsal brain region by a dorsal brain connective (cdbr) (Figs. 4D; 6B). Both parts are connected to the ventral nerve cord (vnc) (Figs. 4A, C; 5E; 6A) by the *cc* (Figs. 4A-C; 5A-C; 6A-C). The latter is composed of two major nerve strands (roots) - the ventral and the dorsal one (visible via serotonin staining, Fig. 4A). The subepidermal *vnc* is composed of two pairs of distinct serotonergic neurite bundles with cell soma concentrated in ganglia (Figs. 5E, G). Anteriorly of the transition from *vnc* to the *cc,* three pairs of neurite bundles (Fig. 4C: pn) emerge from each side of the *cc* and proceed laterally. There they branch off and become part of the intraepidermal peripheral nervous system (Fig. 4C). Anteriorly, a pair of stomatogastric nerve bundles (Figs. 4C; 6A-C) originates medially of the *cc* and fuses with the ventral root of the ventral brain region (Figs. 4B-C; 6A-C). From that point of fusion, two nerves connect a prominent stomatogastric loop-like neurite bundle which proceeds ventrally along the pharynx (Figs. 4C-E; 6A-C). Numerous delicate neurite bundles emerge from that loop-like structure and innervate the anterior stomatogastric system. The dorsal branch of the *vpbr* is connected to the brain via several minute nerves, which run along the prostomium of adult *Hypania invalida* (prn) (Figs. 4B-E; 6A-C).

### Anterior appendages

#### Aulophora larvae of *Lanice conchilega* and adult *Terebella lapidaria* (Terebellidae)

The most prominent structure observable in aulophora larvae of *L. conchilega* are the tentacles at the anterior end (Figs. 2A, B; 3A-D; 6D). The latter are located dorsally to the mouth opening. Cross-sections of the tentacles of adult *Terebella lapidaria* exhibit a prominent longitudinal invagination along the entire tentacle. This prominent structure, which is also present in the tentacles of *L. conchilega,* represents the food rim (see Figs. 3A-C, F), which are not present in the branchiae with their prominent blood vessels (Fig. 3A,E, F). The (buccal) tentacles in *L. conchilega* are innervated by numerous neurite bundles (Figs. 2C, D; 6D, E), which originate in the delicate anlage of the ventral region of the brain (Figs. 2C, E, F; 6E). The ventral region of the brain is connected to both strands (roots) of the *cc* (Figs. 2C-F; 3A; 6D, E). In particular the chaplet-like appearance is obvious in serotonin-like immunoreactivity (Figs. 2C, F; 6D, E). Each anterior appendage is innervated by two pairs of neurite bundles originating from two distinct ganglia located at the base of the tentacle (Figs. 2D, F; 6D, E). The latter nerves run along the entire structure and form a prominent loop at the tentacular tip. Notably, the connection between these ganglia is not direct. Although the respective structures are laying side by side, they are interconnected via the mentioned nerve loop of the tentacles (see Fig. 6E).

#### *Hypania invalida* (Ampharetidae)

Dorsally - at the anterior end of *H. invalida* - four pairs of tentacle-like head appendages with two prominent blood vessels are present (Fig. 5A-D). These appendages are innervated by the first four pairs of lateral neurite bundles, in every case a thick and a slender one, originating from the *vnc* and proceed dorsally (Figs. 4A, C; 6A, C). Along their course - about half way - they are connected to a crossing neurite bundle innervating the lateral organ (Figs. 4A; 6B, C). Afterwards, the lateral neurite bundles terminate in a ganglion (Figs. 4A; 6A, B). From that terminal ganglion, two main branchial neurite bundles emerge and proceed along each branchial structure forming a loop by getting interconnected by numerous ring-shaped neurite bundles (Figs. 4A; 6A).

Additionally, a second type of head appendages – normally exhibited in inverted position inside the pharynx – is present within the oesophageal cavity (see Figs. 4A; 5A-C, 5F-H; 6A, B). These so-called buccal tentacles are innervated by four neurite bundles originating from the ventral and dorsal root of the ventral region of the brain (Figs. 4A-E; 6A-C). These tentacles can be stretched out for food uptake (shown in Figs. 4B, C; 6C).

A combination of all datasets allows for a proximation concerning the most probable location and appearance of these head appendages in everted position and their respective neuronal innervation (Fig. 6C). The assumed position and differentiation between short more proximal tentacles (Figs. 5A, B, H; 6A, C) and long distal tentacles (Figs. 5F-H; 6A, C) is mainly based on the Azan stained-sections and the 3D-reconstruction.

## Discussion

### The brain and major neuroanatomical properties in Terebelliformia

Based on our comprehensive investigations, the terebellid *Lanice conchilega* and the ampharetid *Hypania invalida* possess circumoesophageal connectives (*cc*) which are similarly connected to the brain by two strands (roots), a ventral and a dorsal one. These strands or roots of the *cc* show a clear serotonin-like immunoreactive signal but cannot be seen in Azan-sections. Due to their general location and the patterning in anti-serotonin-staining, we assume them as being homologous to the two roots previous authors described using histological sections [14]. Anatomical differences, in terms of course and extension of the mentioned roots in both investigated taxa, are caused by morphological transitions related to adaptive changes of the entire anterior end in Terebelliformia. Although both roots of the *cc* are closer associated in *H. invalida* than in *L. conchilega*, the transition of the latter into the two paired neurite bundles of the ventral nerve cord is comparable when observing their serotonin-like immunoreactivity. A closer examination of the neuroanatomical characteristics of both taxa shows many similarities in this respect. In anterior direction, the *cc* fuses with the dorsal, more prominent region of the brain. Antero-ventral to the dorsal brain region, two connectives - one splitting from the *cc* (connective of ventral brain region-*cvbr*) and another proceeding from the dorsal brain region (*cdbr*) - fuse by forming the ventral, more slender region of the brain. Thereby, the ventrally oriented brain region splits up distally and forms a distinct ventral and dorsal root, which both innervate the buccal tentacles in *H. invalida* and *L. conchilega*.

Contradictory, earlier investigations focussing on Terebelliformia describe a unified, ribbon-like brain [13] and postulate the ventral and dorsal connective (*cvbr* and *cdbr*) as the two roots of the “common tract” – the latter not being part of the brain. Instead, both roots were described to innervate the lateral part of the “tentacular membrane” and the buccal tentacles.

Nevertheless, our data reveal a protrusion of a slender brain region in ventral direction. This ventral region exhibits two neuronal roots and innervates the buccal tentacles in Terebelliformia. The investigations presented herein demonstrate that nerves of the so-called “tentacular membrane” (see [13]) are in fact loop-like neurite bundles, which connect the dorsal brain region with the dorsal root of the ventral brain region (*prn*). Caused by a hypothesized evolutionary transition of the dorsal lip and associated buccal tentacles from the mouth region towards dorsal in Terebellidae (as summarized in [12]), these prostomial nerves are comparable with those in the ampharetid *H. invalida.* Nevertheless, they are much shorter. The same loop-like nerves innervate the ampharetid buccal tentacles as well as the “dorsal ridge” in Terebellidae (according to [13]) and potentially even the “cephalic veil” in Pectinariidae.

Due to the comparable position of the brain in Terebelliformia [12] and similar innervation patterns – including prostomial loop-like nerves connecting the dorsal to the ventral brain region – our data generally confirm previous assumptions [12–14]. Accordingly, the “cephalic veil” of Pectinariidae, the “dorsal ridge” of Terebellidae and the hood-like “tentacular membrane” of Ampharetidae should be treated as being homologous structures.

In contrast, our conclusions concerning the potential prostomial or peristomial origin of these structures differ from earlier hypotheses. [19] suggested the pectinariid cephalic veil as a fusion of pro- and peristomium. Furthermore, developmental studies support the tentacles of Terebellidae as being part of the prostomium [20], whereas others suggested a peristomial origin of the latter [12]. Only for Ampharetidae developmental and morphological studies both suggest a peristomial origin of the buccal tentacles [12, 15]. Nevertheless, these assumptions concerning the tissue origin of palps in various terebelliform taxa are mainly based on the final position of the latter and the ability to retract the palp-like structures into the mouth opening. Anatomical data including innervation patterns were missing so far.

The question whether the buccal tentacles are of prostomial or peristomial origin can now partly be answered, based on our morphological data. Hence, our comparative investigations do not support a prostomial origin of the tentacles in Terebelliformia. The prostomium and all associated structures are innervated by loop-like nerves connecting the anterior-most ventral and dorsal brain region. Notably, they are limited to the prostomial hood in Ampharetidae as well as the prostomial and very short dorsal ridge in Terebellidae. In contrast, the buccal tentacles in all investigated taxa are associated with the peristomial upper lip and show a differing and much more complex peristomial innervation. Concluding, our data support a peristomial origin of the buccal tentacles in Terebelliformia. A similar pattern can be hypothesized for Pectinariidae, but needs further investigation.

The combination of potentially homologous innervation patterns described for Pectinariidae [13] and our observation concerning terebellid and ampharetid taxa, homologous structures such as the “cephalic veil”, the “dorsal ridge” and the “tentacular membrane” should be defined as prostomial structures.

The earlier interpretation of terebellid tentacles as being of prostomial origin might have been caused by the developmental transition of character complexes involved into the cephalisation processes and obscurities about their ontogenetic origin. This cephalisation is obvious for the ontogenetic transition of branchiae and their inclusion in the formation of the anterior end in Ampharetidae, Alvinellidae, Melinnidae and Terebellidae [6], but is not responsible for the localization of terebelliform tentacles. The latter are characterized by a steady lateral addition of tentacles during ontogenesis. Both processes are independent. Nonetheless, additional detailed morphological as well as comprehensive developmental analyses are necessary for a better understanding of the role of cephalisation and multiplication processes in the formation of morphological features in Annelida.

### Branchiae – anterior transition during cephalisation

The ampharetid *Hypania invalida* bears four pairs of digitate branchiae grouped dorsally on the head, while the two terebellid species *Lanice conchilega* and *Terebella lapidaria* show three pairs of dichotomous branchiae serially arranged along the trunk [6, 21]. The putative sister group of all other Terebelliformia - the Pectinariidae - also exhibit four pairs of branchiae along the segments II-V like shown for many other Terebelliformia [3, 22]. For all species investigated herein, the branchiae were easily identifiable in histological sections by the appearance of an afferent and an efferent blood vessel surrounded by the coelomic cavity.

In Terebelliformia, an ancestral number of four branchiae is assumed, while several reductions, transitions and even multiplication processes took place [6]. Due to developmental studies e.g. [15] the branchiae in larval Ampharetidae occur from segment II-VI and shift towards anterior during ontogenesis. Therefore, branchia in adults are located at segment II and III [6]. All branchial appendages in Terebelliformia are innervated by a more prominent anterior and a slender posterior, segmentally arranged lateral neurite bundle originating from the anterior end of the *vnc*. They proceed in dorsal direction along the trunk musculature and terminate in a branchial ganglion, situated at the base of each branchia. Notably, this observed pattern is comparable to the neuronal innervation pattern known from parapodial appendages in the errant annelids *Neanthes arenaceodentata* (Moore, 1903) and *Platynereis dumerilii* (Audouin & Milne Edwards, 1833) (see [23, 24]). Therefore, an evolutionary scenario including a parapodia-linked origin of the branchial structures and the later involvement in cephalization events has to be assumed. Notably, cephalisation seems to be a widespread evolutionary phenomenon in annelids, and is described for several errant as well as sedentary taxa [25, 26]. Such an ontogenetic transition of larval trunk-associated appendages towards anterior in adult specimens seems to represent an important mechanism. Cephalisation seem to represent one major evolutionary process responsible for the diversity of the sensorial apparatus and even physiological adaptations of the anterior end in Terebelliformia and Annelida in general. Unfortunately, we were not able to compare our observation with the neuronal innervation of the branchiae in adult Terebellidae. The branchiae in the investigated aulophora larvae of *L. conchilega* were still not fully developed and can therefore not be used for detailed interpretations.

### The anterior-most appendages – a multiplications of palps?

In the ampharetid *H. invalida* each tentacle is innervated by four neurite bundles, whereas in total eight prominent neurite bundles proceed along each tentacle in late larvae of *L. conchilega*. However, in both species the tentacles are similarly innervated by neurite bundles, which originate from the ventral and dorsal root of the circumoesophageal connectives. In *H. invalida,* tentacular nerves originate from the ventral root of the more delicate ventral brain region, which is connected to the circumoesophageal connectives (*cc*) by the ventral brain region commissure (*cvbr*). Additionally, they always originate from the dorsal root of the ventral brain region, which is connected to the dorsal, prominent region of the brain by the dorsal brain region commissure (*cdbr*). In *L. conchilega,* nerves of the buccal tentacles originate from neurons arranged arc-like on the ventral region of the brain. They are innervated by neurite bundles of both roots of the *cc*. These neurite bundles split up directly before entering the more prominent dorsal brain region. Such an arc-like set of innervating ganglia (tga) was never described for terebellids so far.

According to various authors, annelid feeding palps are defined as being innervated by nerves originating from the ventral and dorsal main neurite bundles (roots) of the *cc* [13, 14, 19, 27, 28]. As described for the terebellid *Pista cristata* (Müller, 1776) and the ampharetid *Amphicteis* cf. *gunneri* (M. Sars, 1835) [13] a quite similar peristomial innervation pattern of the buccal tentacles can be assumed. Furthermore, they seem closely associated with the nerves of the alimentary canal [13, 14]. By comparing earlier investigations of Orrhage (see [14]) and our results, the branching pattern of all involved neurite bundles is similar. It is shown that stomatogastric nerves only originate from the *cc* or/and its ventral root and thus belong to the ventral brain region. A pattern we can observe in *H. invalida* and *L. conchilega* as well. Accordingly, the ventral strand (root) of the *cc* and the innervation of the entire stomatogastric system are closely associated. A close neuroanatomical connection of the “common tract”/ ventral region of the brain and the stomatogastric neurite bundles seems to be a widespread phenomenon. An alimentary origin of the buccal tentacles is therefore unreasonable based on the presented data.

Furthermore, no obvious developmental connection of the pharynx and the buccal tentacles can be observed for terebelliform taxa so far [15, 17, 20].

According to the current knowledge [13] and our presented data, a similar neuroanatomy can be assumed for Pectinariidae as being the sister taxon of all other Terebelliformia [6]. Many authors also support the homology of the buccal tentacles in all Terebelliformia investigated so far [1, 12, 13]. In conclusion, our data strongly promote a homologization of the buccal tentacles in Terebelliformia with the feeding palps in the remaining Annelida. Accordingly, in Oweniidae (exemplarily shown for *Owenia fusiformis* (Delle Chiaje, 1844) and *Myriowenia sp.* (Hartmann, 1960)), two main neurite bundles innervate the palps or the tentacular crown. These prominent neurite bundles originate dorso-laterally and medio-dorsally from the brain [29]. Together with data from Magelonidae - which show a much more structured brain with a clear interpretation of the palp neurite bundles coming from the ventral and the dorsal region of the brain - a homologization of the feeding palps in Paleoannelida (Oweniidae and Magelonidae) and Terebelliformia is plausible [30] with respect of multiple losses that might have happened in non-sedentarian families. In Sedentaria, comparable palps and palp nerves fulfilling these neuroanatomical criteria can also be found in Orbiniidae [31], Siboglinidae [32, 33], Cirratuliformia [14] and Spionidae/Sabellidae [10, 14, 34]. A detailed comparison of the neuroanatomy of all mentioned taxa highly supports our hypothesis concerning the terebelliform anterior-most head appendages as sharing the same evolutionary (peristomial) origin like the feeding palps of other taxa. Comparable investigations for errant annelids are still pending.

During the ontogenetic formation of the anterior end, a clear differentiation has to be made in respect of “cephalisation” and “multiplication” – not only in Terebelliformia.

In contrast to adult Terebellidae, the aulophora larvae of *L. conchilega* possesses only a few tentacles, which are arranged antero-dorsally on the head. During ontogenesis, multiplication – an increase in the number of similar structures – in this case of the tentacles, leads to a lateral increase of tentacles until adulthood. Inside the tentacular bud, loop-like nerves originate from the ventral brain region and differentiate from lateral in median direction. This multiplication process is also known from other sedentary polychaetes, such as Sabellariidae and Sabellidae [26, 35] and results in an anterior concentration of numerous identical structures, like e.g. feeding palps, which are therefore involved into the formation of the anterior end. In contrast, the anterior clustering of other structures – such as branchiae – is shown to be the result of ontogenetic transition of parapodia-associated structures during cephalisation (see above). Although the result of both processes – multiplication and cephalisation – highly contributes to the formation of the anterior end in annelids (or at least the realisation of the sensorial peculiarities of the head), they have to be considered as independent processes that should be interpreted separately.

## Conclusion

Our comparative and comprehensive approach including various morphological methods and a detailed literature revision sheds new light on the evolutionary origin of anterior head appendages in Terebelliformia.

The brains of the ampharetid *Hypania invalida* and the aulophora larvae of *Lanice conchilega* (Terebellidae) consist of a dorsal, more prominent and a more slender, ventral brain region, which seems to be the result of a brain protrusion. Neurite bundles innervating the buccal tentacles split off from the ventral and dorsal root within the ventral brain region and thus originate from the dorsal and ventral root of the circumoesophageal connectives. In this respect, the observed neurite bundles fulfil the morphological criteria for the innervating neurite bundles of feeding palps known from Palaeoannelida. Furthermore, we confirm a close association of the neurite bundles of the buccal tentacles and neurite bundles of the alimentary tract. Nevertheless, the innervation of parts of the alimentary canal by the ventral strand (root) of the circumoesophageal connectives seems to be the rule and not the exception in Annelida. Thus, we disagree with former conclusions that buccal tentacles are part of the alimentary canal. Accordingly, the buccal tentacles of terebelliform taxa can be homologized with peristomial feeding palps of other Annelida. Hence, multiple losses in non-sedentarian families have to be assumed. Additionally, our data uncover two important and independent processes during the formation and localisation of head appendages in Terebelliformia - cephalisation and multiplication. Hence, both processes result in a concentration of appendages and sensory structures around the head. Unfortunately, there is a huge lack of morphological data concerning these key features of annelid evolution and further investigations are needed to investigate both processes in related families. Furthermore, to gather a comprehensive picture concerning the evolution of head appendages in Annelida in general, additional comparative investigations of anterior appendages in other families are strongly/urgently needed.

## Methods

### Specimen collection

Adult specimens of *Hypania invalida* (Grube, 1860) were collected from the river Rhein near Bonn in summer 2018. Specimens were maintained together with the collected sediment in a freshwater aquarium with a 12 h:12 h light regime at 17 °C at the University of Göttingen. Adult specimen of *Terebella lapidaria* Linnaeus, 1767 were found in crevices on the beach of Le Cabellou, close to the city of Concarneau (Brittany, France) in September 2018.

Aulophora larvae of *Lanice conchilega* (Pallas, 1766) were caught around the island of Helgoland with a plankton net and fixed by employees of the Biological Station Helgoland according to our protocols in autumn 2019.

### Immunohistochemistry

Anatomical details of adult *H. invalida* and aulophora larvae of *L. conchilega* were investigated using standard immunohistochemical staining protocols. Specimens of both species were relaxed in 7% MgCl_2_ and subsequently fixed in 4% paraformaldehyde (PFA) in 1x phosphate buffered saline with Tween (PTW = 1x PBS: 0.05 M PB / 0.3 M NaCl / 0.6% Tween20 (0.4% Tween20 for *L. conchilega*, pH 7,4). Fixation was performed at room temperature (RT) for 2 h for *H. invalida* and 1 h for *L. conchilega*. After fixing, the specimens were washed and stored in PTW containing 0,005% NaN_3_ until usage at 4 °C.

For antibody staining, specimens were rinsed 2 × 5 min in PTW at RT and permeabilized in 10 μg proteinase K/ml PTW (10 min for *H. invalida* and 15 min for *L. conchilega*). After 2 short rinses in glycine (2 mg glycine/ml PTW), and 3 × 5 min washes in PTW, the specimens were re-fixed using 4% PFA in PTW containing 0.6/0.4% Tween for 20 min at RT. Subsequently, the samples were rinsed 2 × 5 min in PTW, 2 × 5 min in THT (0.1 M TrisCl, 0.1% Tween, pH 8,5) and blocked with 5% goat serum (Sigma-Aldrich Chemie GmbH, Steinheim, 25 μl goatserum in 500 μl THT) for 2 h. Afterwards, specimens were incubated with the primary antibodies against α-tubulin (Anti-acetyl α -tubuline, clone 6-11B-1, Merck, Darmstadt, 2 μl tubulin in 500 μl incl. 5% goat serum) and serotonin (5-HT (serotonin), ImmunoStar Inc.,Hudson, USA, 1 μl in 500 μl incl. 5% goat serum) in THT for 48-72 h at 4 °C.

Afterwards, samples were rinsed 2 × 10 min in 1 M NaCl and washed 5 × 30 min in THT.

Subsequently, the samples were incubated in the secondary antibodies goat-anti-mouse 633 (Alexa Fluor® 633 goat-anti- mouse IgG (H + L), Thermo Fisher Scientific Inc., Waltham, USA, 1 μl in 500 μl inlc. 5% goat serum) and goat-anti-rabbit 488 (Alexa Fluor® 488 goat-anti-rabbit IgG (H + L), Thermo Fisher Scientific Inc., Waltham, USA, 1 μl in 500 μl incl. 5% goat serum) in THT for 48-72 h at 4 °C.

After the staining, specimens were rinsed 5 × 30 min in THT and 2 × 5 min in PTW. Additionally, samples were incubated in DAPI (DAPI, Thermo Fisher Scientific Inc., Waltham, USA, 5 μl in PTW) in PTW overnight at 4 °C.

Subsequently, the specimens were dehydrated in an ascending isopropanol series, cleared using Murray’s clear (benzyl alcohol & benzyl benzoate, 1:2) and embedded between two cover slips using DPX mounting medium (Merck, Darmstadt, Germany). The specimens were analysed with a confocal laser-scanning microscope Leica TCS SP8 (Leica Microsystems, Wetzlar, Germany). The confocal image stacks were processed with Leica AS AF v2.3.5 (Leica Microsystems) and Imaris × 64 9.2.1 (Bitplane AG, Zurich, Switzerland).

### Azan staining, histological sections and 3D-reconstruction

For semi-thin sections and AZAN-staining specimens of *Hypania invalida* and *Terebella lapidaria* were processed as described in [36]. Accordingly, specimens were relaxed in 7% MgCl_2_ and then fixed in Bouin’s fluid for 12 h, dehydrated in an ethanol series and incubated in methylbenzoat and butanol. Afterwards the samples were pre- incubated in Histoplast (Thermo Scientific, Dreieich, Germany) and embedded in Paraplast (McCormick Scientific, Richmond, USA). 5 μm thick sections were made using a Reichert-Jung Autocut 2050 microtome (Leica, Wetzlar, Germany). The sections were transferred to albumen-glycerin coated glass slides. Afterwards sections were stained with Carmalaun, differentiated with sodium phosphotungstate (5%), washed in distilled water, stained in aniline blue orange G and subsequently embedded with Malinol (Waldeck, Münster, Germany). In Azan staining, the neuropil of the nervous system stains gray, the nuclei of cell somata stain red, the extracellular matrix stains blue and the musculature stains orange [36]. Each section was digitalized at 40x magnification using a slide scanner (Olympus dotslide (2.2 Olympus, Hamburg)) and aligned using IMOD [37] and imodalign (http://www.q-terra.de/biowelt/3drekon/guides/imod_first_aid.pdf).

**Fig. 7:**
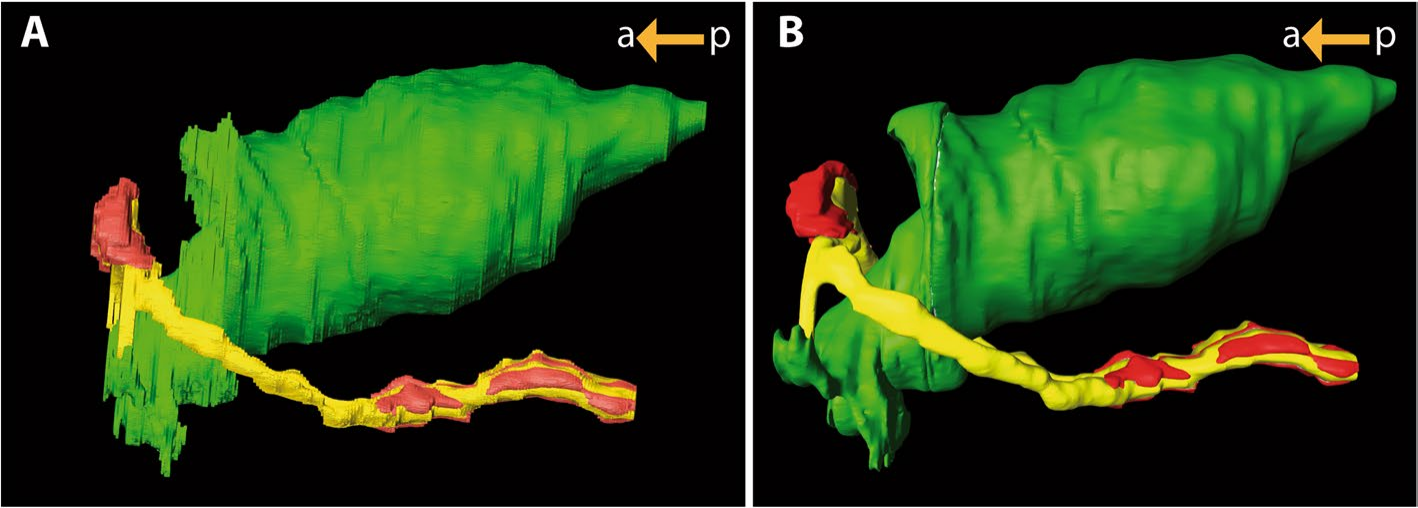
3D-reconstruction based on Azan-stained -sections of *Hypania invalida****.*** **A.** 3D-reconstruction of the brain, circumoesophageal connectives, ventral nerve cord (yellow), cell soma (red) and inverted pharynx and oesophagus (green) using Fiji and Amira. **B.** Same reconstruction using workflow including Blender

For the 3D-visualization we used Amira 2019.1, Meshlab_64bit_fp v2020.6, Blender 2.83 and deep exploration 5.5. Due to the unavoidable artefacts occurring during physical sectioning and the problems coming with it to reconstruct a smooth 3D-model (see Fig. 7A) we used a new combination of freeware resulting in a much more satisfactory result for the eye (Fig. 7B).

## Data Availability

All data analysed in this study are used in figures of this article. The original 3D confocal image stacks can be made available after personal contact with the corresponding authors.

## Abbreviations

*cc*: Circumoesophageal connectives
*cdbr*: Connective of dpbr
*cvbr*: Connective of vpbr
*dpbr*: Dorsal region of the brain
PB: Phosphate buffer saline
PFA: Paraformaldehyde
*prn*: Prostomial nerves
RT: Room temperature
THT: TrisCl buffer
*vnc*: Ventral nerve cord
*vpbr*: Ventral region of the brain
